# Resting-State Functional Connectivity Analyses: Brain Functional Reorganization in a Rat Model of Postherpetic Neuralgia

**DOI:** 10.3390/brainsci12081029

**Published:** 2022-08-03

**Authors:** Shuting Han, Guanzuan Wu, Xiang Wei, Xiaowen Meng, Fengchao Zang, Lan Shen, Hui Dai, Lina Wang, Yonggang Li

**Affiliations:** 1 Department of Radiology, The First Affiliated Hospital of Soochow University, Suzhou 215006, China; tournelsgr@163.com (S.H.); huizi198208@126.com (H.D.); 2 Institute of Medical Imaging, Soochow University, Suzhou 215006, China; 3 Department of Radiology, Shaoxing People’s Hospital, Shaoxing 312000, China; 13402582923@163.com; 4 Department of Endocrinology, Dushu Lake Hospital, Soochow University, Suzhou 215006, China; 20184032027@stu.suda.edu.cn; 5 Department of Endocrinology, The First Affiliated Hospital of Soochow University, Suzhou 215006, China; lingximxw@163.com (X.M.); wangln@suda.edu.cn (L.W.); 6 Jiangsu Key Laboratory of Molecular Imaging and Functional Imaging, Department of Radiology, Zhongda Hospital, Medical School, Southeast University, Nanjing 211189, China; zangfengchao@163.com; 7 Traditional Chinese Medicine Department, The First Affiliated Hospital of Soochow University, Suzhou 215006, China; shenlan19760910@163.com

**Keywords:** neuralgia, postherpetic, chronic pain, magnetic resonance imaging, rats

## Abstract

Postherpetic neuralgia (PHN) is a chronic neuropathic pain syndrome, similar to other chronic pains, the mechanisms of which are not fully understood. To further understand the neural mechanism of this chronic pain and its transition, we performed functional magnetic resonance imaging (fMRI) scans on PHN rat models. Twelve PHN rat models were established by intraperitoneal injection of resiniferatoxin, with an additional 12 rats serving as controls. Nociceptive behavioral tests were performed on these rats and fMRI scans were performed on days 7 and 14 after modeling. Functional connection (FC) analysis was used to investigate the brain FC alterations associated with chronic pain in PHN rats, with the anterior cingulate cortex (ACC) as a seed. Nociceptive behavioral tests showed that PHN rats presented symptoms similar to those of PHN patients. FC analysis showed that compared to the control group, the PHN group showed different FC patterns on days 7 and 14. As can be seen, the brain FC alterations in the rat model of PHN changed dynamically, shifting from brain regions processing sensory information to regions processing emotions and motives.

## 1. Introduction

Postherpetic neuralgia (PHN) is a chronic neuropathic pain syndrome, which is the most common complication of herpes zoster (HZ) [1]. It is defined as pain lasting for more than three months after the HZ rash has healed. According to epidemiological reports, about 25% of people are infected with HZ during their lifetime, and the risk significantly increases after the age of 50 years [2]. Nearly 20% of the patients with HZ will develop PHN, with moderate or severe chronic pain lasting from months to years. Intense pain often affects sleep, mood and work, leading to lower quality of life [3]. PHN imposes a heavy financial burden on society because of the high medical costs of drugs, clinic visits and hospitalizations [4].

Multimodal MRI [5] such as functional magnetic resonance imaging (fMRI) and diffusion-tensor imaging has been applied to study the processing of pain in the human brain, enhancing the understanding of the neurophysiological mechanisms underlying pain conditions. Similar to other types of chronic pain, the mechanism of PHN is not fully understood. There are various theories to explain pain, such as the gate control theory [6] and pain matrix theory [7], involving advanced information conduction and processing, from the simple sensory input to neural network output of the central nervous system. Previous studies [5,6,7,8,9] revealed that PHN was closely associated with structural/microstructural and functional abnormalities in the brain mainly located in the ‘pain matrix’, including the primary somatosensory and secondary somatosensory, the thalamus, insula cortex, parahippocampal gyrus, amygdala, prefrontal cortices, as well as other regions, such as the precuneus, lentiform nucleus and brainstem, significantly affecting multiple dimensions of pain processing, including the sensory, affective, emotional, memory and cognitive aspects [10]. The pain matrix is considered as a characterization of brain regions associated with pain processing. However, the pain-perception network or matrix of chronic pain differs from that of acute pain. Various factors affect the perception of chronic pain in an individual, such as mood, anxiety, attention, past experience and memory. Brain regions pertaining to these factors are also involved in the processing of pain. Moreover, each type of chronic pain has its specific pattern of brain activity [11]. Several longitudinal studies on rodents and humans [12,13,14,15] found that chronic pain was associated with morphological and functional changes in several areas of the brain over time.

Functional magnetic resonance imaging (fMRI) has been performed in PHN patients. However, the results are influenced by various factors, such as the patients’ environment, age and gender. Animal models can avoid the above problems. Previous studies [8,9,16] have shown that a single systemic treatment of resiniferatoxin (RTX) could produce an interesting paradoxical change in thermal and mechanical sensitivities in adult rats, similar to the unique clinical features of patients with PHN. Pan [8] suggested that the diminished thermal sensitivity by RTX could be attributable to the depletion of unmyelinated afferent neurons. Furthermore, the delayed persistent tactile allodynia in RTX-treated rats is likely caused by damage of myelinated afferent fibers and their abnormal sprouting into the spinal lamina II. Although mechanical allodynia and hyperalgesia can be induced by inoculating the hind paws of mice or rats with varicella zoster virus, viral infection models often fail to induce thermal damage and have the disadvantage of tissue inflammation, skin lesions and paralysis due to the spread of the virus in the central nervous system [10]. Therefore, in this study, the RTX model was used as a nonviral PHN model to explore the altered brain networks of this chronic pain condition.

The cingulate gyrus, a part of the limbic system, is one of the components of the medial pain system [17]. The processing of pain information is mainly conducted by the ACC, which is involved in the coding of pain emotion information. In this study, functional connectivity (FC) based on resting-state fMRI was applied in the brain of the RTX-induced PHN rat model with ACC as a seed region, to investigate the underlying central nervous system mechanism of chronic pain and its transitions caused by PHN.

## 2. Materials and Methods

This study was approved by the Animal Care and Use Committee of Soochow University (Project Number 20170185, Approval Date: 27 February 2017).

### 2.1. Animals

A total of 24 male Sprague Dawley rats (weighing 220–250 g) purchased from the Laboratory Animals Center of Soochow University (Animal license No. SYXK Jiang-su 2017-0043), were used in this study. The rats were individually housed in cages with a 12 h light/dark cycle, and free access to food and water. All rats were randomly divided into two groups, the PHN group (*n* = 12) and the control group (Con group, *n* = 12). The PHN group rats were anesthetized with isoflurane (2% in O_2_), and then administered a single intraperitoneal injection of RTX (210 ug/kg, LC Laboratories, Woburn, MA), as previously described [8,9]. RTX was dissolved in a mixture of 10% Tween 80 (Sigma, St. Louis, MO, USA) and 10% ethanol in the normal saline [18]. The CON group was injected with a vehicle, which was dissolved in the same mixture.

### 2.2. Nociceptive Behavioral Tests

The mechanical pain threshold and thermal pain threshold of each rat were measured before RTX or vehicle injection and at different time points after injection (6 h, day 1, day 3, day 5, day 7, day 9, day 11, day 14, day 16, day 21, day 28, day 35 and day 42).

The “up and down” method [19] was used to measure the mechanical pain threshold. After 30 min of adaptation, a series of calibrated von Frey filaments (Stoelting Company, Wood Dale, IL, USA) were applied perpendicularly to the hind plantar surface of the rats on the raised grid floor. Thereafter, sufficient force was applied to cause its slight buckling against the paw, and it was held for about six seconds. Brisk withdrawal or paw flinching was considered as a positive response. The test was repeated three times for each rat, and the average value was calculated.

The thermal withdrawal latency was measured by inducing the latency of rats’ leg reflex with thermal stimulation, as a thermal pain threshold [8,9,18]. After 30 min of adaption, the heat sensitivity was evaluated by a thermal radiation pain meter that exposed the midfoot surface of the hind paw to the light beam on the transparent glass surface. The period of rat paw reflex was recorded and continuously measured three times, and the average value was calculated.

### 2.3. fMRI Examination

A total of 24 male Sprague Dawley rats (weighing 220–250 g) (PHN = 12, CON = 12) were randomly divided into two groups. One group (PHN = 6, CON = 6) was scanned by MRI on day 7 after modeling and the other group (PHN = 6, CON = 6) was scanned by MRI on day 14.

The rats were anesthetized with 5% isoflurane (2% after anesthesia) and a mixture of oxygen and nitrogen (3:7) [20], and subcutaneously injected with 0.05 mg/kg of dexmedetomidine. MRI was conducted on the rats after anesthesia. The head of each rat was fixed with a hook and two ear rods in a prone position on the scanning bed. The heartbeat, respiratory rate and oxygen saturation of the rats were monitored during the scanning. An MRI-compatible device for temperature control and ventilation system was used to maintain the physiological state of the rats.

A 7.0 T animal MRI scanner (Pharma Scan, Bruker Bio spin GmbH, Ettlingen, Germany) with a quadrature surface RF coil was used. Anatomical images were acquired with a turbo-rapid-acquisition relaxation enhancement (RARE) [21] T2-weighted sequence (TR = 3200 ms, TE = 36 ms, FOV = 25 mm × 25 mm, voxel size = 0.065 × 0.065 × 1 mm [3], slice thickness = 1 mm without gap, matrix = 384 × 384, flip angle = 90°, slices = 27). The anatomical images included brain areas from the cerebral olfactory bulb to the caudal region of the cerebellum. A single-shot gradient-echo echo-planar-imaging sequence was used to acquire multiple slices of blood oxygen-level-dependent images (TR = 2000 ms, TE = 19 ms, FOV = 25 mm × 25 mm, voxel size = 0.26 × 0.26 × 1 mm [3], slice thickness = 1 mm without gap, matrix = 96 × 96, flip angle = 90°, slices = 27) [22]. The scan time for the MRI examination was approximately 40 min for each rat.

### 2.4. Data Processing and Functional Connectivity Analysis

Functional data were preprocessed with Statistical Parametric Mapping software (SPM8; Welcome Centre for Human Neuroimaging, UCL Queen Square Institute of Neurology, London, UK) and then entered into the Resting State fMRI Data Analysis Toolkit V1.8 software (REST; State Key Laboratory of Cognitive Neuroscience and Learning, Beijing Normal University, Beijing, China) for analysis. The sequential data-processing steps were as follows: elimination of the first 10 time points, slice-timing adjustment, realignment and correction for head-motion, spatial normalization to the standard rat brain atlas [23], smoothing with an isotropic Gaussian kernel (FWHM = 1 mm), detrending and filtering (0.01–0.1 Hz). Data were excluded if the head motion was more than 1.0 mm in the x, y or z directions or the head rotation was more than 2.0 degrees around any of the three axes.

Region of interest (ROI)-based FC analysis was performed for the ACC region consisting of 9 voxels. The mean time series for the ROI was computed for the reference time course. Thereafter, cross-correlation analysis was conducted between the mean signal change in the ROI and the time series of every voxel in the whole rat brain. Finally, a Fisher z-transform was applied to improve the normality of the correlation coefficients. Both motion parameters resulting from the realignment and the global signal time course were regressed during this analysis to improve the specificity of the FC.

### 2.5. Statistical Analysis

Data of nociceptive behavioral tests were presented as mean ± standard deviation. The effect of RTX on the paw withdrawal threshold and latency was analyzed using Student’s *t*-test. A *p*-value < 0.05 was considered to be statistically significant. For the fMRI data, two-sample *t*-tests were performed to identify the significant changes in FC between the PHN group and the CON group, respectively, on day 7 and day 14 after modeling. Thresholds were set at a corrected *p*-value < 0.001 (cluster size > 20 voxels) using the multiple comparison correction obtained with the AlphaSim method employing Monte Carlo simulation.

## 3. Results

### 3.1. Nociceptive Behavioral Tests

**Figure 1:**
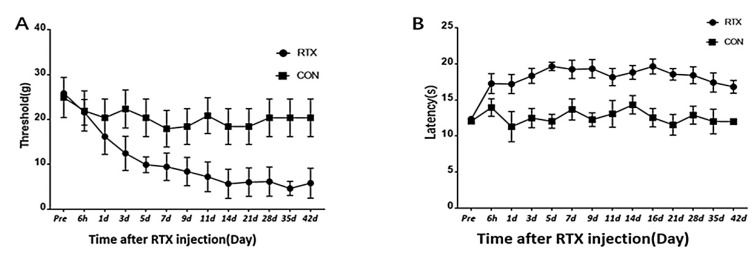
Mechanical pain threshold and thermal pain threshold changes in the PHN and CON groups. (**A**): Changes in the mechanical pain threshold of von Frey filaments in the PHN and CON groups. (**B**): Changes in the thermal pain threshold after thermal stimulation in the PHN and CON groups.

The mechanical pain threshold changes of the PHN group and the CON group were examined, as shown in Figure 1A. After RTX injection, the mechanical pain threshold of rats gradually decreased and stabilized after 14 days, which lasted for at least 42 days (*p* < 0.05, *t* = 4.169). There was no significant change in the mechanical pain threshold of the CON group at different time points.

The thermal pain threshold changes of the PHN group and the CON group were examined, as shown in Figure 1B. After RTX injection, the thermal pain threshold of rats gradually increased and stabilized after five days, which lasted for at least 42 days (*p* < 0.05, *t* = 4.12). There was no significant change in the thermal pain threshold of the CON group at different time points.

### 3.2. Functional Connectivity Analysis

**Figure 2:**
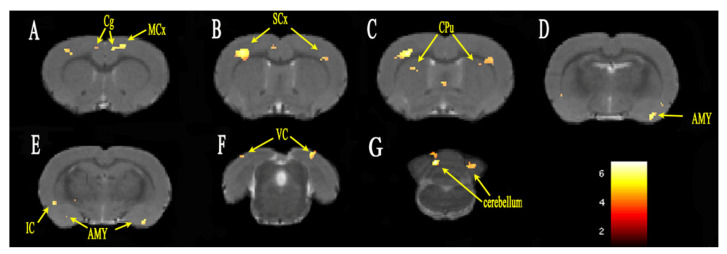
FC alterations in specific brain regions of PHN rats on day 7 (**A**–**G**). ROI heat maps show the brain regions with significantly increased FC. The color scale bar is shown in lower-right part, with corrected *t*-values ranging from 2 to 6. Cg, cingulate gyrus; MCx, motor cortex; SCx, sensory cortex; CPu, caudate putamen; IC, insular cortex; AMY, amygdala; VC, visual cortex; cerebellum.

The group differences of FC analysis with the seed region ACC on day 7 are shown in Figure 2. The FC between ACC and the insular cortex, cingulate gyrus, sensory cortex, motor cortex, amygdala, striatum, cerebellum and visual cortex was significantly increased in the PHN group compared with that in the CON group. The cluster sizes and *t*-values of each brain region are shown in Table 1.

**Figure 3:**
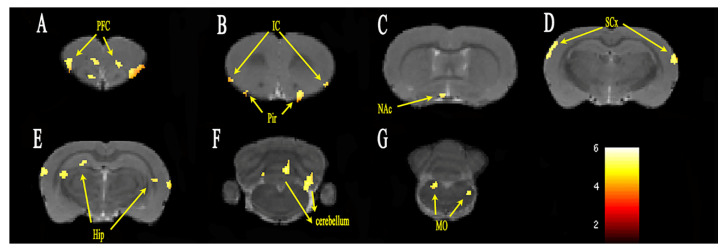
FC alterations in specific brain regions of PHN rats on day 14 (**A**–**G**). ROI heat maps show the brain regions with significantly increased FC. The color scale bar is shown in lower-right part, with corrected *t*-values ranging from 2 to 6. PFC, prefrontal cortex; IC, insular cortex; Pir, piriform cortex; NAc, nucleus accumbens; SCx, sensory cortex; Hip, hippocampus; MO, medulla oblongata; cerebellum.

The group differences of FC analysis with the seed region ACC on day 14 are shown in Figure 3. The FC between ACC and the PFC, insular cortex, hippocampus, sensory cortex, nucleus accumbens (NAc), cerebellum, piriform cortex and medulla oblongata was significantly increased in the PHN group compared with that in the CON group. The cluster sizes and *t*-values of each brain region are shown in Table 2.

## 4. Discussion

A rat model of PHN induced by RTX was used in this study to explore the functional changes in the brain associated with PHN. Unlike other neuropathic pains, patients with PHN often show increased sensitivity to tactile sensation, while thermal sensitivity is reduced. In this study, the tactile sensitivity of PHN rats was significantly increased, while the thermal sensitivity was reduced, which was similar to the symptoms of PHN patients [24]. Although the model did not reflect the pathophysiological process of HZ infection, latency and replication, it appropriately simulated the clinical features of PHN. The changes of mechanical pain threshold and thermal pain threshold in PHN rats were consistent with previous reports [8].

After intraperitoneal injection of RTX, the mechanical pain threshold and thermal pain threshold were significantly changed within 14 days, and then gradually stabilized between day 14 and day 42. Because the pain threshold peaked on day 14, which was indicative of stable pain, day 14 was considered as the time point of chronic pain. The fMRI of the rats was scanned on day 7 and day 14, respectively, corresponding to acute and chronic pain. The FC changes of the whole brain were analyzed, with ACC as the seed. PHN rats showed increased FC between the ACC and sensory cortex, striatum, motor cortex, insular cortex, and cerebellum both on day 7 and day 14, compared with the Con group. Increased FC with the amygdala was observed on day 7 but not on day 14. Meanwhile, on day 14, the FC between ACC and hippocampus, dentate gyrus, NAc as well as PFC (orbital cortex, frontal association cortex, and frontal cortex area 3) were increased, but not on day 7.

Increased FC was observed in the sensory cortex and insular cortex both on day 7 and day 14. The sensory cortex comprises of primary somatosensory and secondary somatosensory components of the lateral pain pathway, and is mainly accepted as the projection of the lateral thalamus nucleus. The neurons in the primary sensory cortex encode the intensity and positional information of the sensation [25], while the secondary sensory cortex has a wide range of receptive fields [26]. The ACC and insular cortex reflect the emotional component of pain as constituents of the medial pain pathway, and they mainly receive projections from the medial nucleus of the thalamus. They are often activated synchronously with a pain stimulation, which may reflect some unpleasant conditions, such as observing aversion, seeing or imagining the pain of other rats [27].

Increased FC was observed in the striatum, motor cortex and cerebellum both on day 7 and day 14. In rodents, the caudate nucleus and the putamen constitute the striatum. Geha [28] found that the activation area of the ventral striatum was decreased after lidocaine therapy in PHN patients. The ventral striatum appeared to be the most responsive to spontaneous pain in PHN. The effect of the striatum on pain processing remains unclear. It may be associated with the neural structures, with a high concentration of endogenous opioids and their receptors, which are involved in the process of endogenous analgesia [29]. The motor cortex is mainly involved in the control of posture. Functional changes of the motor cortex were found in studies of phantom limb pain [30], complex regional pain syndrome [31], and low back pain [32]. The cerebellum is an integrator of multiple effects, including emotional processing, pain regulation, and sensorimotor processing. The cerebellum receives extensive neural inputs from the cortex through the pontine nuclei and the inferior olive nucleus in the brainstem, including several different types of functional inputs associated with pain, such as motion, sensation, visual space, and cognitive information [33]. Helmchen [34] linked the nociceptive activities of the cerebellum to pain, and determined the different modes of activities of the cerebellum for harmless and harmful thermal stimuli. However, due to the complexity of cerebellar function, the specific role of cerebellum in pain perception needs further study.

Increased FC was observed in the amygdala on day 7 but not on day 14 in the present study. As a part of the limbic system, the amygdala is believed to be the integrated processing center of negative emotional and noxious information [35]. Li [36] evaluated the effects of amygdala injury on the early and late stages of injury-induced neuropathic pain model of rats and found that the amygdala was crucial in the early stage of chronic pain. The present study indicated that the amygdala participated in the early stage of PHN, which was in agreement with the study of Li et al.

Additionally, increased FC was seen in the hippocampus, NAc and PFC on day 14 but not on day 7. Hippocampus is closely associated with anxiety, depression, learning and memory, and also plays an important role in context adjustment and regression [37]. Mustso [38] and Ren [39] found molecular and synaptic alterations of the hippocampus in animal models of chronic pain. Apkarian [40] redefined chronic pain as the persistence of pain memory and the inability to eliminate pain memory caused by the initial injury, because learning and memory have an important role in chronic pain. The hippocampus is functionally associated with the NAc and the medial PFC. The NAc is closely related to mood, learning, motivation and addictive behavior, while the PFC is associated with advanced cognition and emotion [41]. Metz [42] and Chang [43] observed functional reorganization of the medial PFC and NAc in the rat model of neuropathic pain, respectively. A one-year longitudinal study of patients with subacute back pain demonstrated that increased FC of the medial PFC and NAc may predict the transition from subacute to chronic pain [44]. The results of the present study were in agreement with these findings. Apkarian [11] believed that different types of chronic pain involve different cortical and subcortical regions, but they all appear to involve different brain regions with time, shifting from brain regions processing sensory information to regions processing emotions and motives. When a body is subjected to a long-lasting noxious stimulus, the NAc and the hippocampus may be preferentially involved, leading to new learning and memory processes. These newly formed pathways may interact with the medial PFC and shift cortical activities from noxious perception to more painful emotional status.

Our findings are also highly consistent with findings on the MRI presentation of PHN patients. A study found that compared with health controls, PHN patients presented increased brain activity in the medial frontal gyrus, precentral gyrus, thalamus and insula and decreased brain activity in the cingulate gyrus and PFC [45]. Increased cerebral blood flow in the striatum, thalamus, insula and left primary somatosensory cortex and decreased CBF in the frontal cortex were observed in PHN subjects [46]. Functional connectivity differences found between acute and chronic pain are associated with sensory and emotional, which closely resemble previous theories of the pain field [47]. Pain is a complex sensory and emotional experience, and different brain regions are implicated in these different aspects of the pain experience.

This study had some limitations. First, the sample size was relatively small, and large-sample-size studies are needed to improve the reliability of the results. Second, the transition from acute to chronic pain in rodents was studied in the present study, but the corresponding phases of pain in rodents may not be applicable to humans. Finally, the rats were anesthetized before immobilization in the scanning coil to suppress movements, which may weaken the FC due to the effect of anesthetics on brain function. It is still helpful to reference rodents’ FC data. Since the rodent brain is not just a scaled-down version of the human brain; its relatively small and flat neocortex shows striking similarities in function and structure, and the subcortical structures have been largely preserved in evolutionary terms [48,49].

## 5. Conclusions

FC changes of the brain areas pertaining to the processing of feelings, emotions, learning and memory in the rat model of PHN may be useful for understanding the pain sensation, mood regulation, learning and memory mechanisms of PHN in humans. The brain FC alterations in the rat model of PHN changed dynamically, shifting from brain regions processing sensory information to regions processing emotions and motives, which may provide an important theoretical basis for the brain function shifting mechanism of PHN.

## Figures and Tables

**Table 1:** The group differences of FC changes between PHN group and CON group on Day 7.

			Coordinates of Peak Voxel		
Brain Region	Cluster Size	*t*-Value	X	Y	Z
L cingulate gyrus	87	5.037	0.4984	1.4022	1.8021
R cingulate gyrus	93	4.029	−0.5641	1.3830	1.3221
L motor cortex	128	5.120	1.4339	1.1642	1.8021
R motor cortex	31	4.065	−3.6445	1.1627	1.8021
L sensory cortex	333	5.021	4.6644	3.8351	0.1221
R sensory cortex	964	6.204	−3.8953	1.8379	0.6021
L amygdaloid body	176	5.107	4.1497	8.8969	−1.3179
R amygdaloid body	86	4.628	−5.7135	9.3718	−3.2379
L striatum	97	3.972	2.6994	7.3334	−2.7579
R striatum	174	4.562	−3.0901	3.6578	0.3621
L poaterior lobe of cerebellum	251	5.621	3.2290	1.9082	−12.1179
R poaterior lobe of cerebellum	217	6.825	−1.1515	3.3053	−14.2279
L corpus callosum	39	4.485	3.4518	2.8256	0.8421
R corpus callosum	52	4.287	−4.8110	3.2258	−0.8379
L visual cortex	547	4.854	3.8444	1.1680	−8.2779
R visual cortex	298	5.059	−4.8357	1.2926	−8.7579
L capsule	41	4.527	2.7060	7.6681	−3.2379
R capsule	81	4.329	−3.7187	6.2112	−2.5179
R insular cortex	59	4.444	−5.8636	6.3370	−2.0379
L anterior lobe of cerebellum	25	5.569	3.0954	1.9005	−12.1179
R bed nucleus of stria terminalis	23	4.122	−1.0756	5.8819	−0.3579
L cerebellar nucleus	94	4.542	2.3166	4.1936	−13.7979
L claustral layer	24	4.725	4.9416	7.5651	−0.5979
R olfactory cortex	99	4.971	−5.7646	2.4493	−9.2379
R pfi flocculonodular lobe	20	5.495	−1.2951	3.1515	−14.2779
L piriform cortex	183	4.693	4.9449	7.5864	−0.8379
R septal area	86	4.543	−0.6812	5.2784	0.1221
L temporal association cortex	20	3.961	6.1131	2.8843	−8.0379
L cerebral peduncle	27	4.506	2.7093	7.6894	−3.4779
L subthalamic nucleus	20	4.497	2.7093	7.5434	−3.4779
L tegmentum of pons	60	4.750	1.221	8.0601	−11.8779

Note: L = left; R = right.

**Table 2:** The group differences of FC changes between PHN group and CON group on day 14.

			Coordinates of Peak Voxel		
Brain Region	Cluster Size	*t*-Value	X	Y	Z
L sensory cortex	234	4.400	7.0997	4.0198	−2.0379
R sensory cortex	365	4.786	−6.7826	1.8627	−3.2379
L insular cortex	317	4.574	4.8987	6.2660	2.5221
R insular cortex	63	3.896	−5.532	4.7447	3.0021
L hippocampus	34	5.225	5.6824	8.3644	−5.8779
R hippocampus	428	4.892	−5.4264	4.1121	−4.6779
L orbital cortex	473	4.702	0.57926	2.6754	5.6421
R orbital cortex	353	4.400	−3.9580	2.8934	5.1621
L frontal association cortex	64	4.603	0.5859	2.5082	5.8821
R frontal association cortex	92	4.431	−3.5604	2.6033	5.4021
L piriform cortex	292	5.483	3.1680	8.1065	2.4021
R piriform cortex	199	4.852	−1.8131	4.5199	4.6821
L anterior lobe of cerebellum	208	5.486	0.6898	4.6820	−12.1179
R anterior lobe of cerebellum	84	4.831	−1.4749	0.8833	−10.1979
L cerebellar nucleus	64	5.131	3.0954	6.1352	−12.1179
R cerebellar nucleus	30	4.306	−2.1101	4.8544	−12.5979
L corpus callosum	52	4.086	5.5554	8.3993	−6.3579
R corpus callosum	31	4.381	−5.8273	4.2350	−4.6779
L dentate gyrus	22	4.479	3.1465	8.0929	−6.1179
R dentate gyrus	93	4.221	−2.4962	2.0278	−3.9579
L medulla oblongata	163	5.314	2.3166	7.8442	−13.7979
R medulla oblongata	257	5.089	−2.5011	6.6474	−13.3179
L poaterior lobe of cerebellum	306	5.700	2.8050	1.7360	−10.4379
R poaterior lobe of cerebellum	301	5.638	−2.808	1.5575	−10.4379
L pfi flocculonodular lobe	46	5.168	3.0954	6.2813	−12.1179
R pfi flocculonodular lobe	47	4.281	−2.1134	4.9791	−12.3579
L tegmentum of pons	63	5.097	2.3133	7.6769	−13.5579
R tegmentum of pons	69	5.040	−2.3675	6.6551	−13.3179
L auditory cortex	359	4.379	6.8489	4.1109	−3.2379
R auditory cortex	256	4.909	−7.1736	2.9256	−3.9579
L olfactory bulb	327	4.653	0.5759	2.6541	5.8821
R olfactory bulb	246	4.707	−1.9467	4.5122	4.6821
L olfactory tubercle	207	5.126	3.1713	8.2739	1.8021
R olfactory tubercle	52	3.979	−0.5707	7.4734	1.8021
R nucleus accumbens	67	4.236	−1.7702	7.4252	1.5621
L anterior commissure	51	4.198	1.6880	6.4974	2.7621
R frontal cortex area 3	24	4.025	−3.8177	2.9438	4.6821
R motor cortex	21	4.080	−3.5571	2.4785	5.1621
R preoptic region	69	4.048	−0.9485	7.7454	0.1221
R septal area	116	4.512	−5.0354	3.4873	−3.9579
R striatum	46	4.026	−1.2224	7.6874	0.6021
L tenia tecta	33	3.823	1.5412	5.6744	3.7221
R third ventricle	73	4.430	−5.2994	3.6391	−4.1979
L fourth ventricle	42	4.777	2.8281	6.7039	−12.1179
L inferior colliculus	26	4.029	3.0492	3.6467	−8.7579
L olfactory cortex	105	5.359	5.8161	8.5181	−5.8779
L visual cortex	24	4.629	2.3876	1.6063	−9.2379

## Data Availability

The datasets used and analyzed during the current study are available from the corresponding author on reasonable request.

## Abbreviations

PHN	postherpetic neuralgia
RTX	resiniferatoxin
fMRI	functional magnetic resonance imaging
Day 7	the seventh day after modeling
Day 14	the 14th day after modeling
FC	functional connection
ACC	anterior cingulate
HZ	herpes zoster
PFC	prefrontal cortices
Thal	thalamus
CON	control
RAGE	turbo-rapid acquisition relaxation enhancement
ROI	region of interest
NAc	nucleus accumbens
Mcx	motor cortex
Scx	sensory cortex
Cpu	Caudate Putamen
IC	insular cortex
AMY	amygdala
VC	visual cortex
Pir	piriform cortex
Hip	hippocampus
MO	medulla oblongata
